# Alterations of DNA damage response genes correlate with response and overall survival in anti‐PD‐1/PD‐L1‐treated advanced urothelial cancer

**DOI:** 10.1002/cam4.3552

**Published:** 2020-10-23

**Authors:** Monika Joshi, Petros Grivas, Amir Mortazavi, Paul Monk, Steven K. Clinton, Michele Sue‐Ann Woo, Sheldon L. Holder, Joseph J. Drabick, Ming Yin

**Affiliations:** ^1^ Penn State Cancer Institute Hershey PA USA; ^2^ University of Washington Seattle Cancer Care Alliance Fred Hutchinson Cancer Research Center Seattle WA USA; ^3^ Division of Medical Oncology Department of Internal Medicine The Ohio State University College of Medicine Columbus OH USA; ^4^ Foundation Medicine Cambridge MA USA

**Keywords:** ATM, bladder cancer, DNA repair, genomic alterations, prognosis

## Abstract

DNA damage response (DDR) gene alterations in cancer are associated with a higher tumor mutational burden (TMB) and may impact clinical outcomes of urothelial cancer (UC). Here, we explore the prognostic role of DDR alterations in advanced UC treated with anti‐PD‐1/PD‐L1 agents. The study included 53 patients who had FoundationOne genomic sequencing and received anti‐PD‐1/PD‐L1 therapy. Fisher exact test and trend test were used to assess differences in objective response rate (ORR). Overall survival (OS) was measured from the time of initial UC diagnosis and Cox proportional hazard regression analysis was performed to calculate hazard ratio (HR) and 95% confidence interval (CI). The cohort had a median age of 66 with 64% receiving platinum‐based chemotherapy. DDR alterations (including *ATM*) were associated with a non‐significantly higher ORR to PD‐1/PD‐L1 blockade (41% vs. 21%, *p* = 0.136). Patients with DDR alterations (excluding ATM) had non‐significantly longer OS, likely due to a small sample size (HR = 0.53, 95% CI 0.20–1.38, *p* = 0.19). *ATM* alterations were associated with a non‐significantly higher ORR (40% vs. 29%, *p* = 0.6), but also with significantly shorter OS (HR = 5.7, 95% CI 1.65–19.74, *p* = 0.006). Patients with ≥ 3 DDR alterations (including *ATM*) had substantially higher TMB (*p* = 0.01) and higher ORR (80%) with PD‐1/PD‐L1 blockade versus 24% ORR in patients with <3 DDR alterations. In summary, DDR alterations were associated with non‐significantly higher ORR and longer OS for patients with advanced UC receiving anti‐PD‐1/PD‐L1 agents. *ATM* alterations were associated with shorter OS.

## Graphical abstract

DDR alterations are associated with higher ORR and nonsignificantly prolonged OS in patients with advanced UC receiving PD‐1/PD‐L1 inhibitors. However, presence of *ATM* alterations correlated with shorter OS, irrespective of a nonsignificant trend towards higher ORR to anti‐PD‐1/PD‐L1 agents.

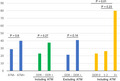

## 1 INTRODUCTION

In spite of recent advances in early diagnosis and treatment, nearly 20% of those afflicted with bladder cancer will die of their disease,^1^ including the vast majority of those presenting with advanced urothelial cancer (UC) involving the regional lymph nodes and distant areas (N+ or M+). The 5‐year mortality remains dismal at 5% for those with distant metastases and 36% for patients that have involved lymph nodes. Median overall survival (OS) for metastatic UC is approximately 15 months.^2^ The advent of newer therapies, such as immune checkpoint inhibitors, targeted therapies, and antibody drug conjugates, has improved the response rates, but there is still urgent need for significant advances and optimal patient selection. There is ongoing research evaluating the role of PD‐L1 expression, tumor mutational burden (TMB), molecular subtyping and DNA damage response (*DDR*) gene alterations, among other biomarkers, as potential predictors of response to anti‐PD‐1/PD‐L1 agents, but no consensus or clinical utility that impacts clinical decision making has yet been reached for the vast majority.^3^, ^4^

Defects in *DDR* genes lead to genomic instability, one of the hallmarks of carcinogenesis, and contribute to malignant progression.^5^ *DDR* gene alterations, in addition to APOBEC mutagenesis, may also contribute to higher TMB. There is a growing body of literature suggesting that *DDR* alterations may have prognostic value in UC. Mutations in *DDR* genes, such as *ERCC2*, *ATM*, *RB1*, *FANCC,* have been correlated with an improved response to cisplatin‐based neoadjuvant chemotherapy^6^, ^7^ perhaps due to the reduced capacity of DDR‐damaged genes to repair cisplatin‐induced DNA strand breaks. More recently, studies suggest that *DDR* alterations are associated with improved response to PD‐1/PD‐L1 blockade in advanced UC;^8^ however, these findings have not been validated in independent cohorts. Interestingly, we previously reported that Ataxia‐telangiectasia mutated (*ATM*) defects correlated with shorter OS in advanced UC.^9^ In this retrospective study, we aimed to expand on earlier findings^8^ and further explore the prognostic role of *ATM* mutations in advanced UC treated with anti‐PD‐1/PD‐L1 agents. We hypothesized that *ATM* mutations would be associated with shorter OS, while other *DDR* alterations would be associated with longer OS in patients with advanced UC treated with anti‐PD‐1/PD‐L1 agents.

## 2 SUBJECTS, MATERIALS AND METHODS

### 2.1 Study population

We collected data from 53 patients from three institutions: (a) Penn State Cancer Institute (b) The Ohio State University Comprehensive Cancer Center (c) Cleveland Clinic Taussig Cancer Institute. The study was approved independently by the institutional review board at all three institutions. Key eligibility criteria include: presence of advanced UC defined as either *de novo* metastatic disease (N_2‐3_ or M_1_) based on the American Joint Committee on Cancer 7^th^ edition or relapsed disease after treatment for localized disease, comprehensive somatic genomic analyses of tumor tissue by FoundationOne, and received anti‐PD‐1/PD‐L1 therapy with palliative intent. We extracted data for disease status, baseline and clinicopathological features, treatment response and outcomes, as well as genomic analyses. Additionally, we collected 38 patients with metastatic UC who did not receive anti‐PD‐1/PD‐L1 treatment as a nonrandomized control population.

### 2.2 Genomic profiling

The methodology for FoundationOne sequencing has been described.^10^ We included both pathogenic variants and variants of unknown significance in our analyses. Tumor mutation burden (TMB) is defined as the total number of nonsynonymous mutations per coding area of a tumor genome [mutations per Megabase (Mb)].^11^ TMB for each patient was provided by FoundationOne and the calculation was based on a targeted panel, as previously described.^12^

### 2.3 Statistical analysis

Chi‐square test was used to test difference in objective response rate (ORR), which was defined as complete or partial response. Cochran‐Armitage trend test was used to determine whether ORR increases along with the number of *DDR* alterations. Nonparametric test (Kruskal‐Wallis Test for three‐group comparison and Mann‐Whitney U test for two‐group comparison) was used to compare mutation counts between groups because of presence of outliers (extreme TMB values). Pearson correlation test was performed to assess the correlation between number of *DDR* alterations and TMB. OS was measured from time of initial UC diagnosis to death of any cause or last follow‐up. Both univariable and multivariable Cox proportional hazard regression analysis were performed to calculate the hazard ratio (HR) and 95% confidence interval (CI), with adjustment of age, gender, race, extirpative surgery of primary tumor, smoking history, relapsed or *de novo* metastasis, and platinum‐based chemotherapy. Kaplan‐Meier curve was used for cumulative probabilities. A number of 28 *DDR* genes selected from FoundationOne gene panel were used for analyses (Table S1). Since *ATM* mutation was a negative prognostic factor in our prior study, we also explored *ATM* and other *DDR* gene alterations separately.

## 3 RESULTS

### 3.1 Patient characteristics

Our cohort of 53 patients had a median age of 66 (range 21–81 years) and a median follow‐up time of 21 months (range: 4.4–138.5 months); 64% of patients had received platinum‐based chemotherapy. As shown in Table 1, there was a predominance of men (66%), white (81%), relapsed (vs. *de novo*) UC (66%), and ever smokers (70%). *DDR* alterations were present in 49% (26/53) patients in our cohort. There was no difference in the proportion of patients receiving prior platinum‐based chemotherapy based on the presence or absence of *DDR* alterations (74% vs. 57%, respectively, *p* = 0.19). We noted that patients who received PD‐1/PD‐L1 treatment had longer OS when compared with 38 patients with metastatic UC (Table S2) who did not receive such therapy (collected separately and used as a nonrandomized control) (HR = 0.43, 95% CI 0.23‐0.82, *p* = 0.01; adjusted HR = 0.35, 95% CI 0.17‐0.70, *p* = 0.003).

**TABLE 1:** Patient characteristics

Parameters	I/O Patients (n = 53) (%)
Median Age (years, range)	67 (21–81)
Gender	
Male	35 (66)
Female	18 (34)
Race	
Non‐white	10 (19)
White	43 (81)
Status of cancer	
*De novo* Metastatic	18 (34)
Relapsed	35 (66)
Smoking	
Never	16 (30)
Ever	37 (70)
Surgery of Primary Tumor	
Yes	32 (60)
No	21 (40)
Platinum‐based Therapy	
Yes	34 (64)^a^
No	19 (36)

^a^ 25 had neoadjuvant or adjuvant chemotherapy.

### 3.2 Association of *DDR* alterations with ORR to PD‐1/PD‐L1 directed therapy

**FIGURE 1:**
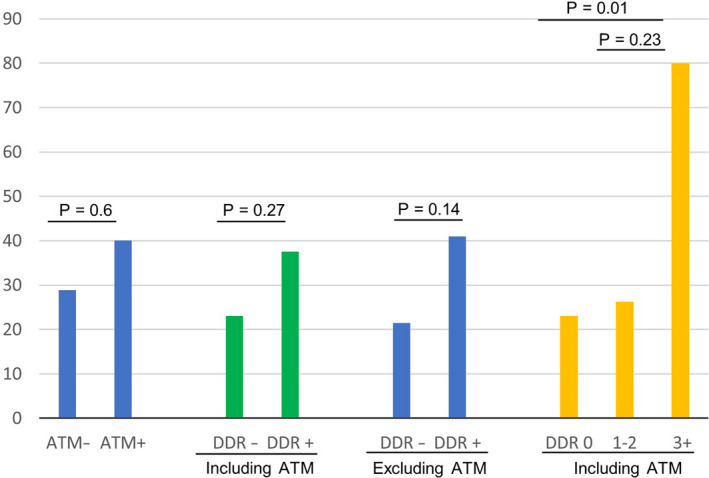
Objective response rate by *DDR* alteration status

Among the 50 patients with available ORR information (assessed by the investigator), we observed a nonsignificantly higher ORR to PD‐1/PD‐L1 blockade in patients who had *DDR* alterations with or without *ATM* (*ATM* included: 38% vs. 23%, *p* = 0.266; *ATM* excluded: 41% vs. 21%, *p* = 0.136) (Figure 1). In addition, patients who had ≥3 *DDR* alterations (including *ATM*) showed substantially higher tumor mutation burden [TMB] [mean 32.5 mutations/Mb (range 14–72) versus mean 12.4 mutations/Mb (range 0.9–78), *p* = 0.01]. Most importantly, these patients experienced a higher ORR to PD‐1/PD‐L1 blockade compared with patients with <3 *DDR* alterations (80% vs. 24.4%; *p* = 0.024). Overall, there was a trend for higher ORR with the presence of higher number of *DDR* alterations (including *ATM*) to PD‐1/PD‐L1 inhibitors (*p*_trend_ = 0.02).

### 3.3 Association of individual pathways with ORR to PD‐1/PD‐L1 directed therapy

**FIGURE 2:**
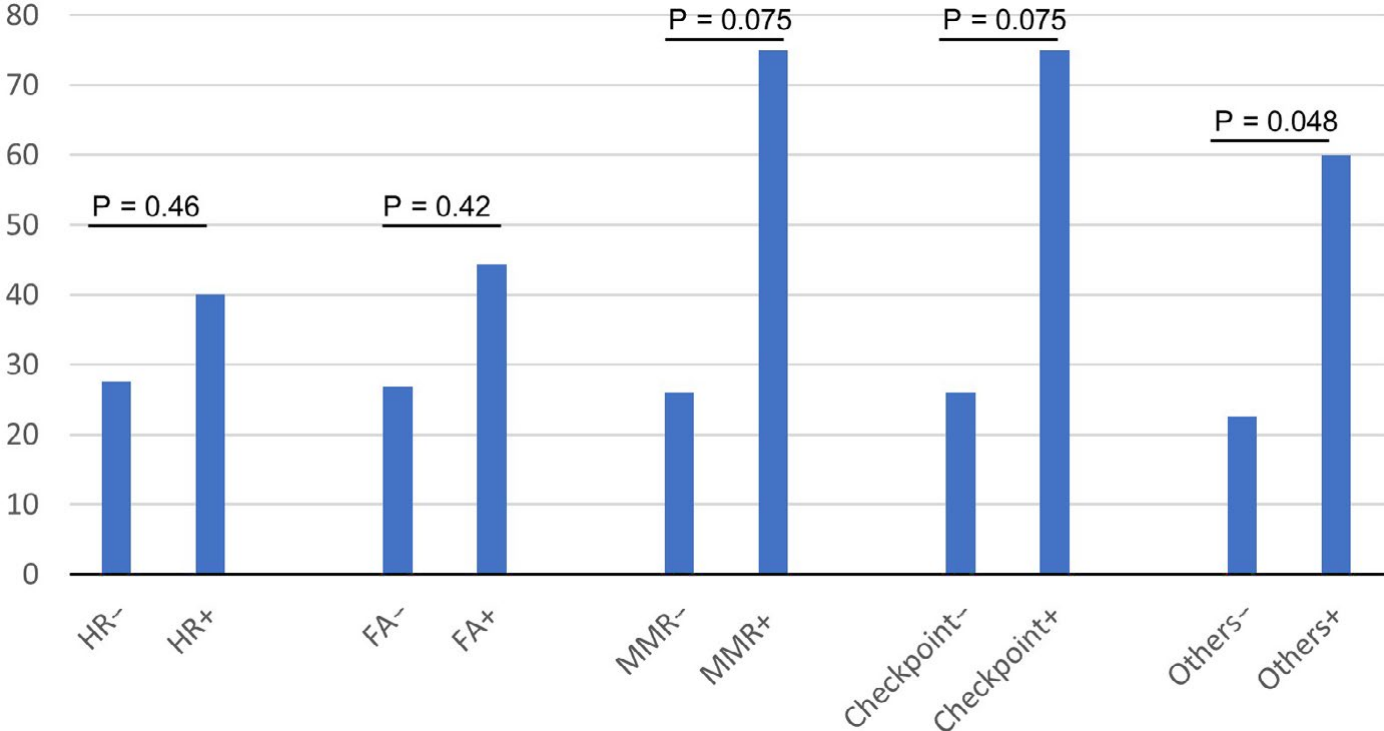
Objective response rate by alteration of *DDR* pathways

We grouped available *DDR* gene alterations into five different pathways, including: homologous recombination (HR) (altered in 10 patients), Fanconi anemia (FA) (9), mismatch repair (MMR) (4), checkpoint (4) and “all others” (10). Alterations in all five *DDR* pathways seemed to correlate with higher ORR, which was most pronounced in MMR, checkpoint and “all others” pathways (Figure 2).

### 3.4 Association of *DDR* alterations with overall survival to PD‐1/PD‐L1 directed therapy

**FIGURE 3:**
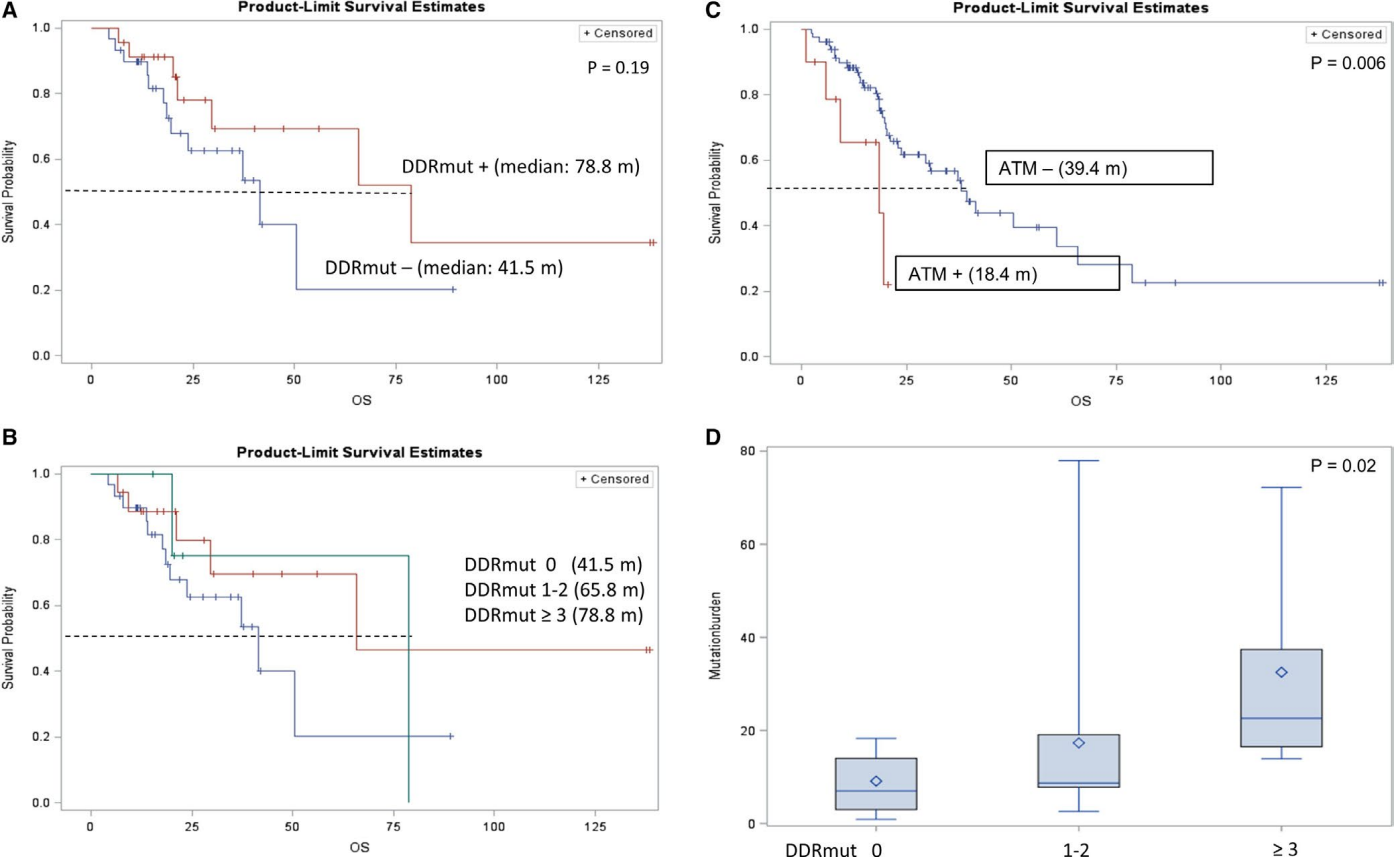
Association of clinical outcome with DDR among patients who received PD‐1/PD‐L1 inhibitors. 3A: OS based on presence or absence of *DDR* alterations; 3B: OS based on number of *DDR* alterations; 3C: OS based on presence or absence of *ATM* alterations; 3D: Tumor mutational burden based on *DDR* status

Patients with *DDR* alterations (including *ATM*) showed similar OS with those without *DDR* alterations (HR = 0.93, 95% CI 0.37–2.34, *p* = 0.88). After multivariable analysis with adjustment of age, gender, race, surgery, smoking history, relapsed or *de novo* metastasis and platinum treatment, the conclusion was not changed (adjusted HR = 0.88, 95% CI 0.34–2.27, *p* = 0.79). However, patients with *DDR* alterations (excluding *ATM*) seemed to have longer OS, although significance was not reached (HR = 0.53, 95% CI 0.20–1.38, *p* = 0.19; adjusted HR = 0.49, 95% CI 0.18–1.38, *p* = 0.18) (Figure 3A and Table 2). In addition, we also observed a trend towards longer OS with increasing number of *DDR* alterations (Figure 3B); median OS: 41.5 for 0 *DDR* versus 65.8 for 1–2 *DDR* versus 78.8 months for ≥3 *DDR*), but statistical significance was not reached (*p* = 0.33).

**TABLE 2:** Associations between ATM/other DDR (oDDR) alterations and OS

	N	Univariable			Multivariable^a^		
		HR	95% CI	*p*	HR	95% CI	*p*
ATM Alterations							
No	47	1			1		
Yes	6	5.7	1.65–19.7	0.006	9.3	1.99–43.2	0.005
oDDR Alterations							
No	30	1			1		
Yes	23	0.53	0.2–1.38	0.19	0.49	0.18–1.38	0.18
oDDR Alteration Number							
0	30	1			1		
1–2	18	0.49	0.17–1.42	.19	0.45	0.15–1.37	0.16
≥ 3	5	0.67	0.15–3.1	.61	0.49	0.18–1.38	0.72

^a^ Adjustment by age, gender, race, surgery, smoking history, relapsed or de novo metastasis and platinum treatment.

### 3.5 Association of *ATM* alterations with clinical outcomes to PD‐1/PD‐L1‐directed therapy

In our present cohort we observed 11% (6/53) of patients harboring *ATM* alterations in the tumor. We were unable to demonstrate the difference between deleterious mutations and variants of unknown significance given the small number of patients. Patients with *ATM* alterations seemed to favor higher ORR to PD‐1/PD‐L1 blockade (ORR, 40% vs. 28.9%, *p* = 0.6), but the difference was not significant **(**Figure 1 **).** Additionally, similar to our previous findings we noted that the presence of *ATM* alterations was associated with significantly shorter OS (HR =5.7, 95% CI 1.65–19.74, *p* = 0.006; adjusted HR =9.3, 95% CI 1.99–43.2, *p* = 0.005) in the overall cohort (Figure 3C and Table 2), as well as in the subgroups with and without platinum‐based chemotherapy (platinum group: HR = 5.27, 95% CI 0.95–29.36, *p* = 0.058; adjusted HR = 16.6, 95% CI 1.66–165.7, *p* = 0.017; nonplatinum group: HR = 6.46, 95% CI 1.05–39.76, *p* = 0.044; adjusted HR = NA).

### 3.6 Association of *DDR* gene alterations with TMB

TMB data were available for 34 patients. We found that *ATM* alterations were associated with higher TMB, although the large variability in TMB precluded detecting a statistically significant impact (mean TMB: 25.4 for *ATM* alteration vs. 13.6 for *ATM* wild type, *p* = 0.62). We observed a trend of higher TMB with increasing number of *DDR* gene alterations (mean TMB: 8.6 for 0 *DDR* vs. 16.4 for 1–2 *DDR* vs. 32.5 for ≥3 *DDR*) (Figure 3D); this difference was significant by non‐parametric testing (*p* = 0.02).

### 3.7 Association of TMB with clinical outcomes of PD‐1/PD‐L1 treatment

**FIGURE 4:**
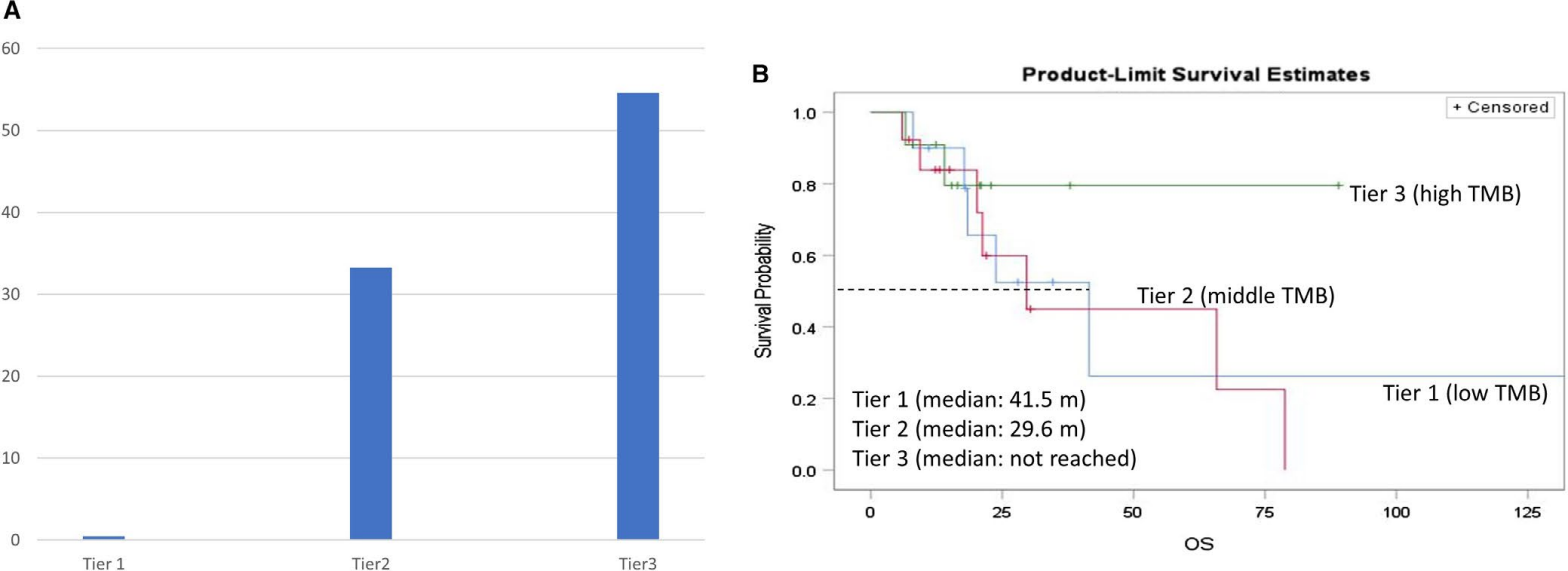
Association of clinical outcome with tumor mutation burden (TMB). 4A: Objective response rate based on TMB groups; 4B: OS based on TMB groups

To determine whether TMB may play a role in mediating treatment response and outcomes, we split patients into 3‐tiers (low, intermediate and high) based on TMB and found that ORR was 0% versus 33.3% versus 54.6% in the three groups, respectively (*p* = 0.04). There was a moderate but significant correlation between the number of *DDR* alterations and tiers of TMB (correlation coefficient = 0.39, *p* = 0.023). The Kaplan‐Meier curve showed that the 3rd‐tier patients (with high TMB) seemed to have the longest OS (Figure 4), while patients of 1st and 2nd tiers did not have significantly different OS. Statistical significance was not reached in the comparison between the 3rd‐tier and the other two tiers by Cox regression analyses (HR = 0.44, 95% CI 0.1–1.98, *p* = 0.28; adjusted HR = 0.4, 95% CI 0.07–2.29, *p* = 0.31).

## 4 DISCUSSION

There is an imperative need to identify prognostic and predictive biomarkers in the era of personalized medicine. Alterations in *DDR* pathways may play an important role in urothelial carcinogenesis.^13^, ^14^ *DDR* pathways are critical for maintenance of genomic integrity and alterations in these genes can enhance the carcinogenesis cascade. Several *DDR* pathway inhibitors have shown promising efficacy in multiple tumor types and are currently under development.^15^, ^16^, ^17^, ^18^, ^19^ There are multiple *DDR* pathways, such as HR, FA, MMR, and NER, which play important roles in the development of cancer and are being targeted for therapeutic development. An earlier report suggested that the presence of any *DDR* alteration was associated with higher ORR to checkpoint inhibitor therapy (67.9% vs. 18.8%; *p* < 0.001).^8^ Even though our sample size is modest, we were able to confirm prior findings. We demonstrated that in patients with advanced UC treated with PD‐1/PD‐L1‐agents the presence of *DDR* alterations was associated with a trend towards longer OS. Moreover, there was a trend for higher ORR with increased number of *DDR* alterations (*p* = 0.02), while patients with ≥3 DDR alterations (including *ATM*) benefited the most from PD‐1/PD‐L1 blockade. Furthermore, we were able to show a favorable ORR for alterations in each of the five DDR pathways, particularly in MMR, checkpoint and “all others” pathways. We observed that patients’ tumors with ≥3 *DDR* alterations tended to have substantially higher TMB, while those patients appeared to benefit the most from PD‐1/PD‐L1 blockade compared with patients with tumors having <3 *DDR* alterations.

*ATM* is a well‐recognized tumor suppressing gene located on chromosome 11q 22‐23, in the family of PIKK genes and is activated with double DNA strand breaks. Activation of ATM results in phosphorylation of p53 and cell cycle checkpoint arrest (CHK2), resulting in G1/S cell cycle arrest via CDC25A and Cyclin‐CDK (Cyclin‐dependent kinase) complexes.^20^ Deleterious mutations and deletions in *ATM* are common across multiple cancer types, and the presence of *ATM* alterations in metastatic/advanced UC is a poor prognostic biomarker, irrespective of platinum‐based treatment.^21^ Similar to those results, we were also able to show these findings in this PD‐1/PD‐L1‐treated UC cohort. However, it is intriguing to note that the presence of *ATM* alterations was associated with a nonsignificant trend for higher ORR to PD‐1/PD‐L1 blockade. Thus, with OS seemingly poor in multiple cancer types with *ATM* alterations regardless of immunotherapy or platinum‐based treatment,^9^ one could assume that patients with tumors harboring *ATM* alterations would need a novel therapeutic approach for improving outcomes. Perhaps treating this subset of patients with ATM, ATR or PARP pathway‐directed targeted therapy, with or without immunotherapy, could be a potential therapeutic approach to be explored in clinical trials.^22^, ^23^

TMB has been considered a potential biomarker predictive of response to immune checkpoint inhibitors across tumor types, including UC.^8^, ^24^ Cancer cells with higher TMB may have more neoantigens, and therefore be more likely to respond to immunotherapy. Defect in *DDR* genes can contribute directly to higher TMB (along with the well‐known APOBEC mutagenesis).^25^ We showed a correlation between DDR defects and TMB in advanced UC, which could result from increased genomic instability. TMB was higher in patients with increased number of *DDR* alterations, which correlated with ORR. These results were consistent with findings from Teo *et al*.^8^ *ATM* alterations seemed to be associated with higher TMB, which may explain the numerically (but not significantly) higher ORR to PD‐1/PD‐L1 therapy in patients with *ATM* altered tumors. *ATM* alterations may have harmful effects facilitating tumor progression due to functional loss beyond its role in DNA repair. Indeed, ATM plays multiple roles in cancer biology.^26^ Its functional loss has been implicated in accelerated EMT and poor prognosis in other malignancies.^27^ We are currently performing further cellular and animal experiments to understand possible underlying mechanisms.

There are a number of limitations of our study. First, we were only able to use OS, instead of cancer‐specific survival (CSS) or progression‐free survival (PFS), for endpoint comparison because CSS/PFS information was not available in our dataset; however, OS is still a meaningful and clinical relevant endpoint. Secondly, due to the retrospective nature of the study, patients may not have received uniform treatment and may not have had the same type and interval of tumor assessments. Although we tried to control confounding factors with multivariable analyses, a number of factors, such as Bajorin or Bellmunt risk scores, were not collected and adjusted for. Moreover, there may have been variability in the timing and site of tumor sample collection. The captured genomic alterations may possibly reflect posttreatment changes, and therefore cannot be used as predictive biomarkers, based only on this study. Furthermore, we included any *DDR* alterations in our analyses without differentiating alteration type, mono‐ versus bi‐allelic status, germline versus somatic, and potential functional impact (e.g. deleterious mutations, variants of unknown significance). However, Teo *et al*. showed that both *DDR* alterations of “unknown significance” and of deleterious nature may have prognostic value in patients with advanced UC treated with anti‐PD1/PD‐L1 agents.^8^ The small sample size, in conjunction with potential selection and confounding biases (including the nonrandomized cohort), render our study results only hypothesis‐generating. Moreover, the 28 DDR genes contained in FoundationOne test may not cover a complete list of genes critical in DNA damage response, although they come close. This could be considered an inherent study limitation. Lastly, PD‐L1 status assessment, alterations in other critical *DDR* genes, such as NER pathway, genome‐wide loss of heterozygosity, homologous recombination deficiency, molecular subtypes, microsatellite instability, and other putative biomarkers were not evaluated in this study.

## 5 CONCLUSIONS

*DDR* alterations are associated with higher ORR and nonsignificantly prolonged OS in patients with advanced UC receiving PD‐1/PD‐L1 inhibitors. We observed higher TMB in tumors with ≥3 *DDR* alterations and the higher ORR in these patients to PD‐1/PD‐L1 inhibitors could possibly be also due to higher TMB. However, in line with our previous findings, presence of *ATM* alterations correlated with shorter OS, irrespective of a nonsignificant trend towards higher ORR to anti‐PD1/PD‐L1 agents, suggesting the possibility that these patients may require additional novel therapeutic approaches. Further studies are needed to assess the clinical utility of *DDR* alterations in directing therapies in UC.

## CONFLICT OF INTEREST

Monika Joshi has research grant through Big Ten Cancer Research Consortium from AstraZeneca and Pfizer/EMD Soreno. Petros Grivas has done consulting for AstraZeneca, Bayer, Bristol‐Myers Squibb, Clovis Oncology, Driver, EMD Serono, Exelixis, Foundation Medicine, GlaxoSmithKline, Genentech, Genzyme, Heron Therapeutics, Janssen, Merck, Mirati Therapeutics, Pfizer, Roche, Seattle Genetics, QED Therapeutics; previously participated in an educational program for Bristol‐Myers Squibb; his institutions have received research funding from AstraZeneca, Bavarian Nordic, Bayer, Bristol‐Myers Squibb, Clovis Oncology, Debiopharm, Genentech, GlaxoSmithKline, Immunomedics, Kure It Cancer Research, Merck, Mirati Therapeutics, Oncogenex, Pfizer, QED Therapeutics (all unrelated in the last 3 years). Amir Mortazavi serves in advisory board of Seattle Genetics, Janssen, Debiopharm Group. Ming Yin is a stockholder of Novavax, Inc. All others have no conflicts of interest relevant to the subject matter or materials discussed in the manuscript.

## AUTHOR CONTRIBUTION

Conceptualization: Ming Yin, Monika Joshi and Petros Grivas, Collection of the data: Ming Yin, Monika Joshi and Petros Grivas, Statistical analysis: Ming Yin and Monika Joshi, Writing–original draft: Ming Yin, Writing–review and editing: Ming Yin, Monika Joshi, Petros Grivas, Amir Mortazavi, Paul Monk, Steven K Clinton, Michele Sue‐Ann Woo, Sheldon L. Holder and Joseph J. Drabick.

## Supporting information

Table S1‐S2

## Data Availability

Data available by request.
