# Supplementary material for: Alterations of DNA damage response genes correlate with response and overall survival in anti‐PD‐1/PD‐L1‐treated advanced urothelial cancer
Source: Cancer Med. 2020 Oct 23;9(24):9365–72. doi: 10.1002/cam4.3552 (PMC7774722; doi:10.1002/cam4.3552)
Supplement: Supplementary file 1 — Table S1‐S2 [file CAM4-9-9365-s001.docx]

| **SUPPLEMENTARY TABLE 1**  **Selected DNA damage response genes** | | | | | | | | |
| --- | --- | --- | --- | --- | --- | --- | --- | --- |
| **HR** |  | **FA** | | **Checkpoint** | | **MMR** |  | **Others** |
| BRCA1 |  | BRIP1 |  | CHEK1 |  | MLH1 |  | POLD1 |
| BRCA2 |  | FANCA |  | CHEK2 |  | MSH2 |  | POLE |
| MRE11 |  | FANCC |  | ATM |  | MSH6 |  | BAP1 |
| RAD50 |  | FANCF |  | ATR |  | PMS2 |  | BARD1 |
| RAD51 |  | FANCG |  |  |  |  |  | CDK12 |
|  |  | FANCL |  |  |  |  |  | MUTYH |
|  |  | FANCD2 |  |  |  |  |  |  |
|  |  | PALB2 |  |  |  |  |  |  |
|  |  | BLM |  |  |  |  |  |  |

| **SUPPLEMENTARY TABLE 2**  **Patient characteristics** | |
| --- | --- |
| **Parameters** | **Non I/O Patients**  **(n = 38) (%)** |
| **Median Age (years, range)** | 64 (41 – 91) |
| **Mean TMB (/Mb, range)** | 15.5 (2.4 – 63.6) |
| **Gender** |  |
| **Male** | 30 (79) |
| **Female** | 8 (21) |
| **Race** |  |
| **Non-white** | 12 (32) |
| **White** | 26 (68) |
| **Status of Cancer** |  |
| ***De novo* Metastatic** | 12 (32) |
| **Relapsed** | 26 (68) |
| **Smoking** |  |
| **Never** | 14 (37) |
| **Ever** | 24 (63) |
| **Surgery of Primary Tumor** |  |
| **Yes** | 22 (58) |
| **No** | 16 (42) |
| **Platinum-based Therapy** |  |
| **Yes** | 23 (61) |
| **No** | 15 (39) |
| **DDR Alteration** |  |
| **Yes** | 23 (61) |
| **No** | 15 (39) |
